# Fully Digital Fabrication of Maxillary Obturator by Enhancing Rough Scan Data With Blender®: A Case Report

**DOI:** 10.7759/cureus.85698

**Published:** 2025-06-10

**Authors:** Aleksandar Naydenov, Rumen Radev, Svetoslav Slavkov, Ivan Gerdzhikov

**Affiliations:** 1 Prosthetic Dental Medicine Department, Faculty of Dental Medicine, Medical University - Sofia, Sofia, BGR; 2 Cranio-Maxillofacial Clinical Department, University Multiprofile Hospital for Active Treatment and Emergency Medicine “N.I. Pirogov", Sofia, BGR

**Keywords:** 3d printing, cad, digital dentistry, intraoral scanning, obturator

## Abstract

Obturators are prosthetic devices designed for patients with maxillofacial defects resulting from surgery, trauma, or congenital conditions. Traditional methods for obturator fabrication are often labor-intensive and uncomfortable for the patient, requiring multiple appointments and manual impressions. This case report presents one of the first fully digital workflows for the design and fabrication of an Aramany class 2 obturator using intraoral scanning, digital design, and 3D printing, without the need to use any type of impression materials and working models. This novel approach proved efficient, cost-effective, and provided optimal patient comfort.

## Introduction

Obturators have long been used to restore functional and esthetic deficits in patients with maxillary defects. Traditionally, their fabrication involves a manual impression and laboratory procedures, which are time-consuming and may cause discomfort for patients with sensitive post-surgical or traumatized tissues. Recently, digital technologies have opened new pathways for streamlining the process, with advantages such as enhanced patient comfort, reduced appointment times, and sufficient adaptation to the prosthetic field. There is not enough information in the currently available literature describing the digitalization of the process, and all the available research includes semi-digital approaches where either a conventional impression was taken or there was a need for fabrication of a working model. This case report describes a fully digital workflow in the fabrication of an obturator for a patient with a post-surgical maxillary defect, highlighting the techniques, tools, and the advantages and disadvantages observed during the treatment.

## Case presentation

Patient background

**Figure 1:**
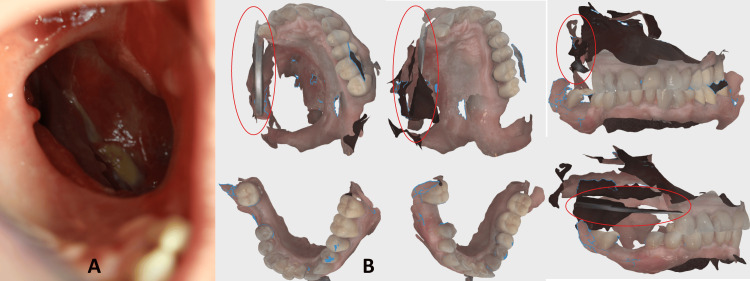
A) The oro-antral communication is visible before the fabrication of the obturator. B) Intraoral scans of the lower and upper jaw. Mirror retracting the cheek is circled with red.

A 65-year-old female patient presented with a partial maxillectomy defect following resection surgery as treatment for squamous cell carcinoma. The resection line reaches the middle line of the hard palate and distally extends to the soft palate. The patient reported experiencing severe challenges related to maintaining proper speech, chewing, and swallowing, as well as a severe disturbance in facial esthetics, post-surgery. The patient is not going under any kind of treatment, including medication and radiation (Figure 1).

Clinical and radiographic examination

An intraoral examination revealed a well-defined surgical defect in the left posterior part of the maxilla, with no signs of infection or inflammation.

Clinical steps and laboratory steps

Maxillary solid bulb obturator was proposed as a prosthetic treatment solution in this Aramany class 2 defect. Given the patient’s discomfort with traditional impression techniques, a fully digital approach was selected to fabricate the obturator. The workflow involved the following steps.

Intraoral scanning

The defect area and remaining maxillary structures, as well as the lower jaw, were scanned using an intraoral scanner (Virtuo Vivo, Straumann Group, Basel, Switzerland), capturing accurate 3D details without the need for a physical impression. Because of the mobility of the cheeks and soft tissues around the defect, we used the dental mirror’s handle to retract the cheeks as much as possible. Due to its tetragonal form, the mirror’s handle was easily recognized by the intraoral scanner software. Without using this approach in previous times, we had a lot of superimpositions of the images, and the final image lacked critical data. The patient tolerated the procedure well, reporting minimal discomfort.

Computer-aided design (CAD)

The stereolithography (STL) files (upper jaw, lower jaw, bite) were imported into Blender 4.1. (Blender Institute, Amsterdam, Netherlands), where the following operations were applied:

• Smooth modifier: Smooths the mesh by flattening the angles between adjacent surfaces in it, just like the smooth mode in edit mode. It smoothens the mesh without subdividing it; the number of vertices remains the same.

• Connect surfaces: Method 1: Select both the edges and press the F key to join them by creating a new surface. Method 2: Select the vertices/or edges you want to merge, then press ALT + M and choose one of the options displayed [1].

• Fill holes: Three different options were used here: A) The "Bridge Edge Loops" tool. With two edge loops selected, it will create a new surface in the middle of the two loops. B) The "Fill" tool. With two surfaces selected, it will create a new surface in the middle of the two faces. C) The "Extrude" tool to create a new surface. With an edge or a surface selected, it will create a new surface that is perpendicular to the selected element [2].

**Figure 2:**
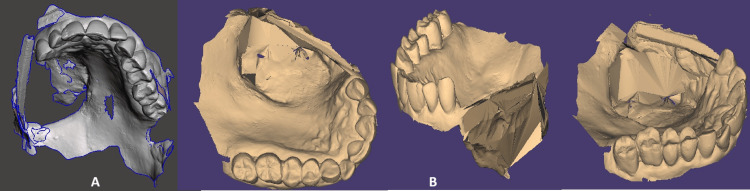
A) Stereolithography (STL) file from scanning the upper jaw, with unnecessary information cut; B) Images from a few aspects after adjustments in Blender. It is visible that most of the holes are closed, and the projection of the oro-antral communication is closed.

With the described procedures done to modify the intraoral scan, we resulted in an STL file, which does not have deformations and is ready to be uploaded as a prosthetic base into exocad (exocad GmbH, Darmstadt, Germany)(Figure 2).

**Figure 3:**
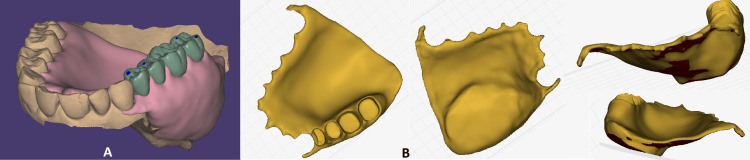
A) View of the obturator design in exocad. The base of the obturator is pink, and the teeth are green. B) The base of the obturator is visible in "Alpha 3D" software (Alpha 3D, Tallinn, Estonia) used for preparing files before 3D printing.

In the CAD software exocad - Rijeka 1 (exocad GmbH, Darmstadt, Germany), a virtual obturator was designed, conforming to the anatomy of the defect while providing adequate support for the oral soft tissues. Customization of the obturator shape ensured stability and enhanced comfort. The partial denture mode in exocad was used. The teeth and base of the obturator were designed in two separate files. The base was designed so that it penetrates 3-4 mm into the defect, giving the hollow bulb form of the obturator. The design also compensates for the lack of both hard and soft tissue, giving full support to the cheek (Figure 3).

3D printing

The final obturator model was printed using biocompatible, dental-grade resins (Premium Teeth HT A2 Resin (Form 4)) used for teeth and Dental LT Comfort Resin (Form 4) used for the base and the bulb) on a 3D printer (Form 4B) (Formlabs Inc., Somerville, MA). The printing process took approximately three hours, followed by post-curing and finishing.

Trial fitting and adjustments

**Figure 4:**
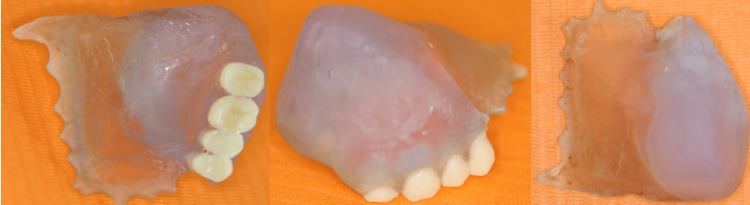
Obturator base and teeth adhered to each other, seen from different aspects.

During a subsequent appointment, the obturator was fitted intraorally. Minor adjustments were made to ensure optimal fit and comfort. The patient reported improved retention and was pleased with the esthetic outcome. The adaptation period took two weeks (Figure 4). 

Patient follow-up

**Figure 5:**
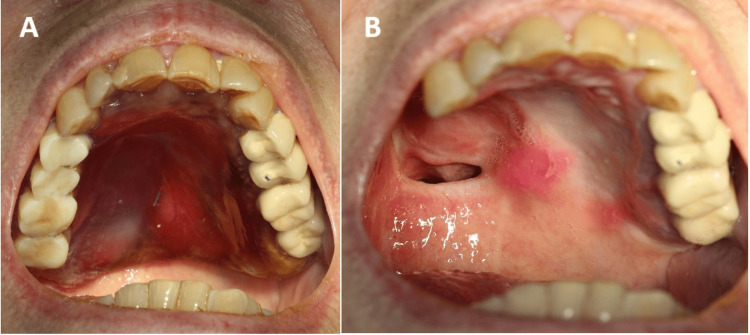
A) Intraoral view of the obturator after nine months. B) Intraoral view of the oro-antral communication. It is visible that it is well-shrunk. The pink appearance on the soft palate, at the right border of the defect, is a small portion of adhesive used for better retention.

A nine-month follow-up showed no adverse reactions or discomfort. The patient reported functional improvements in speech, swallowing, and esthetics. Minimal adjustments were made, and the patient expressed satisfaction with the reduced number of appointments and the comfort of the digital impression. Intraorally, it was observed that the size of the defect was significantly reduced, probably due to hypertrophy of soft tissue (Figure 5).

## Discussion

Obturators have a long history. They were first described in the 16th century by Paré [3]. Traditionally, the fabrication of an obturator has involved several labor-intensive steps, including multiple impressions, model casting, wax-up, and try-ins, which not only consume considerable clinical and laboratory time but also cause discomfort for the patient. Since the 16th century, the technique for fabrication and design has evolved. The contemporary approach includes digitalization of the process, but still, in the literature, there is no case described as a fully digital approach.

We have searched PubMeD, Web of Science, and CrossRef with the following keywords: “obturator”, “3D”, “virtual”, “digital”, and combinations between them. The outcome of our research was that we did not find even a single publication for a fully digital approach in the fabrication of an obturator. Still, there are a few publications that describe semi-digitalization of the process. Most of the found authors describe that they have used silicone impression materials at some point during the fabrication process. In most of the cases first the clinician takes silicone impression and after that it is scanned to digitize the gypsum cast or the silicone impression directly [4], In small amount of cases fully digital approach for obtaining data of the prosthetic field was used but the fabrication of the obturator after that is conventional like in the case reported by Ünal et al. [5].

Park reported for intraoral scanning of the defect and then virtually designing a metal framework, after which they had taken a silicone functional impression. In their study, it is visible that the defect is not scanned fully, and a lot of information about the prosthetic field is missing [6].

There are also cases where the clinician uses cone beam computed tomography (CBCT) image to extract the needed data and to create a custom tray, but still uses manual impression material as a continuation in the workflow for fabricating an obturator [7].

Khalaf et al. report that after one year, digitally fabricated obturators are highly retentive and have excellent tissue surface adaptation upon fabrication. They also report that after application of load, a reduction of retention and lack of tissue adaptation resulted [8].

If milling is used instead of 3D printing, the assembly of base and teeth is eliminated, producing a monolithic obturator [9].

Murat and Batak report for the elimination of problems that could arise from conventional techniques, such as distortion, tearing, or detachment of the impression material during removal from a maxillary defect. Moreover, he concluded that digital techniques decreased the impression steps and were accepted by a patient suffering from dental anxiety [10].

The previously cited case reports report that minimizing the number of clinical visits needed reduces the burden on the patients, particularly those with complex medical histories or high levels of anxiety.

Digital scanning is a faster, less invasive procedure compared to conventional impression methods, significantly improving patient comfort. Additionally, because the digital workflow is quicker, patients can receive their obturators faster, helping to address any speech or swallowing difficulties more promptly. This leads to higher overall patient satisfaction, which is especially important for individuals with maxillofacial defects, as these patients often face significant psychosocial challenges due to altered facial aesthetics and functional limitations.

By reducing the need for elastomeric impression materials, stone casts, and traditional wax setups, the digital approach is not only more efficient but also cost-effective and environmentally friendly [11].

It eliminates certain lab procedures, such as casting and model fabrication, which traditionally require substantial time and manual labor. The streamlined workflow benefits both clinicians and dental technicians by reducing the potential for cross-contamination and the need for complex infection control measures. This efficiency also has the potential to make obturator fabrication more accessible and affordable, therefore benefiting a broader patient population [12].

A few limitations and considerations should also be addressed. As a result of the mobility of the soft tissues and lack of specific structures to identify, superimpositions have occurred, resulting in poor quality of the scanning. We suggest the usage of small intraoral patches, which could adhere to the soft tissues bordering the defect; they have the potential to enhance the process of scanning because the intraoral scanner can recognize them more easily than the soft tissues alone.

The intraoral scan file is not suitable to be used directly in the specialized dental software for virtual design (exocad)and requires additional virtual adjustments beforehand. The method that we used to improve the quality of the STL file can lead to imprecision in the process of closing all the empty spaces and reproducing virtually the missing tissues. An experienced professional is needed to perform the virtual adjustment of the raw file in Blender [13].

The advancements of digital technologies, such as artificial intelligence (AI) and machine learning, offer increased possibilities for further enhancing the accuracy and efficiency of obturator fabrication. AI algorithms, for example, could assist in detecting specific anatomical features or compensate for difficult scanning areas, potentially improving accuracy even in challenging cases.

Advances in CBCT combined with AI segmentation can possibly lead to designing an obturator without even taking any kind of impression intraorally. Additionally, advances in material sciences, particularly in 3D printing, are likely to result in obturator materials that offer better biocompatibility, durability, and aesthetics.

## Conclusions

Fully digital workflows in obturator fabrication offer significant advantages over traditional methods and enhance semi-digital methods. This approach promotes patient comfort and allows for accurate, rapid fabrication of the obturator. The digital fabrication of obturators should be considered a viable alternative in clinical cases where time efficiency and patient comfort are essential. It is important to find a material that can adhere to saliva and be easily recognized by the intraoral scanner to reduce the images’ overlapping and to improve accuracy in the final scan. Further researches in this topic are needed to confirm our conclusions.
